# Exploring nursing interventions in family-based approaches for preventing bullying among children and adolescents: a scoping review

**DOI:** 10.1186/s12912-025-03221-7

**Published:** 2025-05-27

**Authors:** Iyus Yosep, Ai Mardhiyah, Helmy Hazmi, Rohman Hikmat

**Affiliations:** 1 Department of Mental Health, Faculty of Nursing, Universitas Padjadjaran, Sumedang, Jawa Barat, Indonesia; 2 Department of Nursing, Faculty of Medicine, University of Malaysia Sarawak, Kota Samarahan, 94300 Malaysia; 3 Nursing Department, Faculty of Health Science, Universitas ‘Aisyiyah Bandung, Bandung, Indonesia

**Keywords:** Adolescents, Bullying, Children, Family, Interventions, Preventing

## Abstract

**Background:**

Bullying is a prevalent issue faced by children and adolescents in both school and community settings. One contributing factor to bullying behavior is the limited involvement or inadequate role of the family in addressing this issue. However, there is a noticeable gap in existing research regarding nursing interventions that focus on family-centered approaches to prevent bullying. Identifying and analyzing these interventions is crucial to enhancing the role of families in reducing bullying behavior. The aim of this study is to explore and map the various nursing-led, family-based interventions designed to mitigate bullying among children and adolescents, and thereby addressing this critical scientific gap.

**Methods:**

This study employed a scoping review method following Arksey and O’Malley’s framework, including identifying research questions, systematically searching CINAHL, PubMed, and Scopus. The databases used are CINAHL, PubMed, and Scopus. The major keywords used are parenting, family interventions, bullying, adolescents, and children. Inclusion criteria in article selection were family-based intervention, English language, full-text, original research, and publication period in the last 15 years (2010-2024). Data analysis was carried out descriptively qualitatively.

**Results:**

The results of this research show that there are 10 articles that discuss Interventions focused on Family to reduce bullying behavior in children and adolescents. Various types of Interventions focused on Family are effective in reducing bullying behavior in children and adolescents, including improving parenting patterns, family-based education, and collaboration between school and family (p value < 0.05). Some of the activities carried out are education, role play, counseling, managing conflict, and conducting assessments. Interventions can be carried out offline and online. Interventions focused on Family offer a holistic and sustainable approach to dealing with bullying behavior in children and adolescents. The advantages of this intervention include improving family relationships, strengthening communication skills, and better understanding of children’s emotions.

**Conclusions:**

This study highlights the critical role of families in addressing bullying and underscores the need for holistic interventions involving families, schools, and communities. Clinically, nurses can support families by providing education and strategies to build resilience in children facing bullying.

## Introduction

Bullying is the action of a person or group of people that causes other people to feel persecuted, intimidated, afraid, and the victim is powerless to prevent this behavior [1]. The act of bullying cannot be separated from the power gap between the perpetrator and the victim so that the victim feels disadvantaged, oppressed, or hurt [2]. Bullying behavior can also be carried out either directly or indirectly or through social media [3]. Bullying is intentional and repeated behavior that occurs over a period, involving an imbalance of power between the perpetrators and the victims [4].

The results of research conducted in five Asian countries stated that Indonesia was in first place in the incidence of bullying in schools with a percentage of 83%. Survey results show that the number of reports of bullying incidents in schools reached 40% and 32% of them reported experiencing physical violence [5]. Bullying is a major phenomenon throughout the world. The prevalence of bullying is estimated at 8 to 50% in several Asian, American and European countries [6]. The results of research conducted by the National Association of School Psychologists show that more than 160,000 teenagers in the United States miss school every day because they are afraid of being bullied [7].

Bullying has been linked to increased risks of anxiety, depression, low self-esteem, and even suicidal ideation, with long-term consequences persisting into adulthood [8]. Furthermore, research suggests that bullying does not only impact victims but also bystanders and perpetrators, contributing to a cycle of aggression and maladaptive social behaviors [9]. Given these significant implications, it is crucial to explore effective intervention strategies that address bullying comprehensively. Studies have shown that family involvement plays a pivotal role in mitigating bullying behavior and fostering resilience in children [10]. Therefore, conducting research on anti-bullying interventions, particularly those involving family participation, is crucial to developing comprehensive strategies that can mitigate these negative effects and promote a safer school environment.

The impact of bullying has a wide range of impacts, including physical, psychological and social for victims and perpetrators. Physically, victims of bullying often experience physical injuries such as bruises, wounds, or even damage to nerve tissue due to violence committed by the perpetrator [11]. The psychological impacts include anxiety disorders, depression, low self-esteem, and can even cause eating and sleeping disorders [12]. In addition, victims of bullying can also experience decreased academic performance due to difficulty concentrating and persistent anxiety [13]. Socially, they may experience social isolation, have difficulty building healthy interpersonal relationships, and sometimes experience stigma from peers or society [14]. On the other hand, bullies also experience negative impacts, including the risk of being involved in criminal behavior in adulthood and difficulty building good relationships with other people due to a lack of empathy and adequate social skills [15].

Factors that cause bullying behavior can come from factors within the individual and factors from outside the individual. These factors can include personality factors, interpersonal communication between teenagers and their parents, the role of peers, and school climate [16, 17]. Previous study show that the most important factor in children’s involvement in bullying behavior is family factors [15]. This is in line with previous studies which state that parenting or conditions during childhood are one of the triggers for the development of aggressive patterns in children [18, 19]. Previous research also shows that bullying behavior is related to parental styles that use physical punishment, spending less time with adults, and having poor family functioning [20]. Apart from family factors which play a role in the formation of bullying behavior, there are factors which also play an important role, namely factors within the individual, for example empathy, shame and locus of control are often implicated as determining factors in the role of bullying [21].

Nurses play a crucial role in family-based interventions for preventing bullying among children and adolescents. As healthcare professionals who are often in direct contact with both children and their families, nurses have the opportunity to educate and empower families to address bullying behavior proactively [22]. Nurses can help identify risk factors for bullying within the family dynamic, such as communication patterns, parenting styles, and environmental influences, and provide guidance on positive parenting practices that promote empathy, respect, and non-violence [23]. By facilitating family discussions and providing tailored interventions, nurses can strengthen the family unit’s ability to recognize and address potential bullying behaviors early on. Additionally, nurses are in a unique position to collaborate with schools, communities, and mental health professionals to create a comprehensive support system for families [24].

Parenting is the way parents carry out their role, especially in educating their children, starting from making rules, teaching values/norms, and love [25]. One thing that influences parenting is the living environment [26]. According to previous study show that the family environment, especially parents, is a factor that has a stronger influence on the incidence of bullying behavior compared to other environments [27]. This research explains that there is a significant relationship between parenting styles and aggressive behavior in adolescents. Triggers for bullying behavior are strongly influenced by various factors. There are 7 factors that trigger bullying behavior, namely class differences, seniority traditions, conflict within the family, disharmonious school situations, individual/group character, and wrong perceptions. on the victim’s behavior [28].

Despite the existing evidence emphasizing the importance of family and parenting in influencing bullying behavior, there remains a lack of comprehensive exploration of nursing-led, family-based interventions specifically aimed at preventing bullying among children and adolescents. While meta-analyses have highlighted the significance of family and parent training in reducing bullying, they often focus on general approaches without delving into structured, targeted interventions facilitated by healthcare professionals, particularly nurses. Furthermore, previous studies tend to address isolated aspects of family influence, such as parenting styles, without systematically mapping how these approaches can be operationalized into evidence-based practices. This gap underscores the need for a scoping review to synthesize existing knowledge, identify effective family-based strategies, and provide a foundation for future nursing practices and policies aimed at bullying prevention. By addressing this gap, this study will contribute to developing a more holistic understanding of the role of nursing interventions in empowering families to mitigate bullying behavior in children and adolescents.

## Materials and methods

### Design

**Fig. 1:**
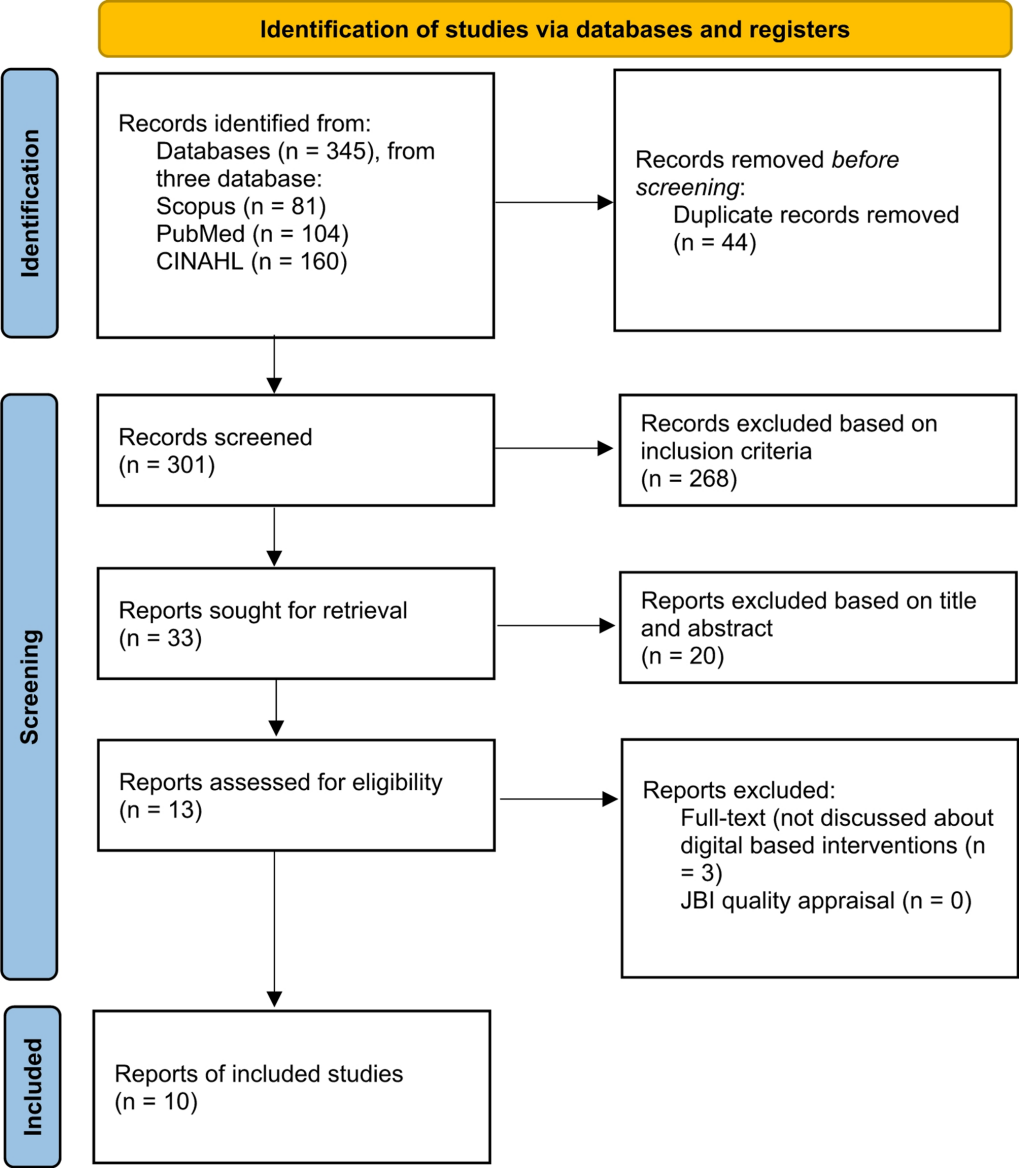
PRISMA flow diagram

The authors used a scoping review design. The stages followed in the study design using the scoping review approach, according to Arksey and O’Malley, involve five main steps [29]. First, determining the objectives and scope of the research, which includes identifying specific research questions and the scope of the literature to be investigated. Second, identifying relevant studies using a systematic and detailed search strategy. Third, selecting studies that comply with the predetermined inclusion and exclusion criteria. Fourth, extracting data from the selected studies using appropriate data extraction tools. Fifth, writing, mapping, and interpreting the study results to present relevant findings comprehensively. The authors used the PRISMA Flow Diagram guidelines for article selection from the initial research results of three databases: CINAHL, PubMed, and Scopus (Fig. 1) [30].

### Search strategy and eligibility criteria

The databases used include Scopus, PubMed, and CINAHL to obtain various articles from large databases that provide various research on health. Article searches were conducted in January-February 2024. Keywords used may include general terms such as are parenting, family interventions, bullying, adolescents, and children. Searches in each database use search conditions such as the use of Mesh Terms and Boolean operators. The keywords used are (“Parenting“[Mesh] OR parenting OR parent* OR family) AND (“Family Therapy“[Mesh] OR “Family Intervention*” OR “Family Support”) AND (“Bullying“[Mesh] OR bullying OR cyberbullying OR “peer victimization”) AND (“Adolescent“[Mesh] OR adolescent* OR teen* OR youth OR “young people”) AND (“Child“[Mesh] OR child* OR “school-aged” OR pediatric). The research question in this scoping review is “What types of nursing-led, family-based interventions have been implemented to reduce bullying behavior among children and adolescents?” searching strategy in this study were:

Scopus: (TITLE-ABS-KEY(“parenting” OR “parent*” OR “family”) AND TITLE-ABS-KEY(“family therapy” OR “family intervention*” OR “family support”) AND TITLE-ABS-KEY(“bullying” OR “cyberbullying” OR “peer victimization”) AND TITLE-ABS-KEY(“adolescent*” OR “teen*” OR “youth” OR “young people”) AND TITLE-ABS-KEY(“child*” OR “school-aged” OR “pediatric”) AND TITLE-ABS-KEY(“nursing intervention*” OR “nursing care” OR “nursing strategy” OR “nursing practice”))

PubMed: ((“Parenting“[Mesh] OR parenting OR parent* OR family) AND (“Family Therapy“[Mesh] OR “Family Intervention*” OR “Family Support”) AND (“Bullying“[Mesh] OR bullying OR cyberbullying OR “peer victimization”) AND (“Adolescent“[Mesh] OR adolescent* OR teen* OR youth OR “young people”) AND (“Child“[Mesh] OR child* OR “school-aged” OR pediatric) AND (“Nursing“[Mesh] OR “nursing intervention*” OR “nursing care” OR “nursing strategy” OR “nursing practice”))

CINAHL: ((MH “Parenting” OR parenting OR parent* OR family) AND (MH “Family Therapy” OR “Family Intervention*” OR “Family Support”) AND (MH “Bullying” OR bullying OR cyberbullying OR “peer victimization”) AND (MH “Adolescents+” OR adolescent* OR teen* OR youth OR “young people”) AND (MH “Child+” OR child* OR “school-aged” OR pediatric) AND (MH “Nursing Care” OR “nursing intervention*” OR “nursing strategy” OR “nursing practice”))

### Inclusion and exclusion criteria

The authors used PCC’s framework, namely Population: adolescents; Concept: nursing-led family-based intervention; Context: reducing bullying behavior. The screening process was conducted by two independent authors who assessed each retrieved record based on the predetermined inclusion criteria. If there were discrepancies or disagreements during the screening process, they were resolved through discussion. In cases where a consensus could not be reached, a third author was consulted to make the final decision. This process ensured the reliability and validity of the study selection. Inclusion criteria include studies that focus on anti-bullying interventions involving the family as an integral part of the intervention strategy. We included studies published in English to ensure accessibility to widely recognized and peer-reviewed literature, facilitating consistency in analysis. Only full-text, original research articles were considered to ensure methodological rigor and the inclusion of primary data. The publication period was limited to the last 15 years (2010–2024) to capture recent developments, emerging trends, and contemporary best practices in family-based anti-bullying interventions while maintaining a sufficient pool of studies for analysis. Exclusion criteria include studies that do not specifically address family interventions in reducing bullying behavior among adolescents, studies available only in abstract form, and gray literature such as dissertations, conference proceedings, and reports, as these sources may not have undergone rigorous peer review.

### Data extraction

The data extraction process is a critical stage in research that involves collecting important information from various sources. Manual tables were used to summarize key elements such as authors, research objectives, country of origin, intervention methods, instruments used, samples taken, and research results. This structured approach facilitates comprehensive analysis by allowing researchers to identify patterns, trends, and implications across the reviewed studies. Before full data extraction, a pilot test was conducted on a subset of articles to refine the extraction process, ensure consistency, and resolve any ambiguities in categorizing data. This step helped to improve the reliability of the extraction procedure and align the research team’s understanding of the data points.

To ensure accuracy and consensus in data extraction, each article was reviewed independently by two authors. In cases where differences in interpretation arose, discussions were conducted to reach a consensus. If disagreements persisted, an independent expert in the field was consulted to provide an objective assessment. However, during this study, no significant disagreements required expert consultation, as consensus was reached through discussion among the research team.

### Quality appraisal

The Joanna Briggs Institute (JBI) instrument for quality appraisal in scoping reviews aims to assist researchers in evaluating the methodological quality of the studies they include in scoping reviews [31]. The JBI instrument for quality appraisal in a scoping review consists of a series of questions or criteria designed to assess the methodological quality of the studies included in the scoping review. These criteria can include aspects such as study design, clarity of objectives, data collection methods, data analysis, as well as suitability between research questions and the design and methodology of the study being conducted. The instrument used in the RCT design consists of 13 statements and the quasi experimental design consists of 9 statements. Answer options consist of yes, no, and not applicable. Answers of yes are given a score of 1 and answers of no and not applicable are given a score of 0. The minimum score limit in the JBI instrument is above 75% as the threshold for the JBI score for articles in this research. The Joanna Briggs Institute instrument was used to assess the quality of selected studies. Quality assessment was carried out by two authors who are experts in their fields. If differences of opinion occur, in-depth discussions are held to reach an agreement. If significant differences of opinion remain, consultation with an independent expert may be undertaken to decide on a final assessment.

### Data analysis

Data analysis was carried out descriptively qualitatively using a content analysis approach. This process aimed to identify and describe emerging themes from the extracted research findings, providing a comprehensive understanding of the effectiveness of family-focused interventions in reducing bullying behavior among adolescents. After thoroughly reviewing the articles and analyzing the extracted data, the authors classified intervention types based on similarities in their approaches and implementation. Data analysis was conducted independently by two authors to ensure accuracy and reliability. In cases where differences in interpretation arose, discussions were held to reach a consensus. During this study, all disagreements were successfully resolved through discussion, and it was not necessary to involve a third author for arbitration.

## Results

The results of initial research from three databases, namely CINAHL, PubMed, and Scopus, the authors found that there were 345 articles. After that, the authors carried out elimination based on duplicate articles, there were 44 duplicate articles. then, the authors carried out elimination based on the inclusion criteria, there were 268 articles that did not match the inclusion criteria determined by the researcher and 20 articles that did not match the title and abstract. Then, the authors read the full-text articles, the authors found that there were 3 articles that were not in accordance with the research objectives and did not discuss family-based intervention to prevent bullying behavior in children and adolescents. The authors found that there were 10 articles that met the criteria determined by the authors. The authors carried out a quality appraisal of the 10 articles, all articles had a JBI score above 75% (Table 1).

**Table 1:** JBI critical appraisal tool

References	JBI critical appraisal tool	Study design
(van Niejenhuis et al., 2020) [32]	84,6% (11/13)	RCT
(Healy & Sanders, 2014) [25]	100% (13/13)	RCT
(Roberto et al., 2017) [35]	84,6% (11/13)	RCT
(Burkhart, 2012) [36]	92,3% (12/13)	RCT
(Tosun & Mihci, 2020) [38]	92,3% (12/13)	RCT
(Kim et al., 2021) [39]	84,6% (11/13)	RCT
(Díaz-Caneja et al., 2021) [40]	84,6% (11/13)	RCT
(Lester et al., 2017) [33]	92,3% (12/13)	RCT
(Cross et al., 2018) [34]	92,3% (12/13)	RCT
(Farmer et al., 2017) [37]	84,6% (11/13)	RCT

Based on the characteristics of articles from various countries, namely 1 article from the Netherlands [32], 3 articles from Australia [25, 33, 34], 2 articles from the USA [35, 36], 1 article from New Zealand [37], 1 article from Turkiye [38], 1 article from China [39], 1 article from Spain [40]. The respondents in this study varied, involving teachers, parents, and children or teenagers. Respondents to the 10 articles all involved parents. The range of respondents in this study was 72-3200 respondents. All articles use an RCT design. The authors found that there are three types, namely cooperation between schools and families, family-based education, and improving parenting patterns. Activities in this intervention include education, role play, counseling, managing conflict, and conducting assessments. Intervention can be carried out offline and online (Table 2).

**Table 2:** Extraction data

No	Author(s) & year	Country	Aims/purpose	Sample size	Design	Intervention type & duration	Outcomes & measurement tools	Key findings
1.	(van Niejenhuis et al., 2020) [32]	Netherlands	To improve parent-school cooperation in counteracting bullying	Teachers (*n* = 83), Parents (*n* = 153), Children (*n* = 2,510)	RCT	Parent-school cooperation intervention (1 school year)	**Questionnaire**: Revised Olweus’ Bully/Victim Questionnaire **Outcome**: Improved parent-teacher cooperation, but no significant improvement in competence to address bullying (*p* = 0.03).	Intervention led to increased parental and teacher engagement in anti-bullying efforts.
2.	(Healy & Sanders, 2014) [25]	Australia	To determine the impact of a family intervention on victimization and emotional distress in children	111 families with children aged 6–12 experiencing chronic bullying	RCT	Resilience Triple P Family Intervention (3 months)	**Questionnaire**: Preschool Peer Victimization Measure (PPVM), Parent-Child Discussion Task (PCD) **Outcome**: Significant reduction in victimization and emotional distress (*p* < 0.01).	Family intervention effectively reduced bullying victimization and improved children’s emotional well-being.
3.	(Roberto et al., 2017) [35]	USA	To evaluate the impact of cybersecurity education on bullying prevention	51 parents of middle school students	RCT	Cyberbullying Prevention Program (Duration not specified)	**Questionnaire**: Attitudes and Predicting Social Behavior **Outcome**: Significant reduction in bullying behavior (*p* < 0.05).	Parents became more aware of online risks and reported reduced cyberbullying incidents.
4.	(Burkhart, 2012) [36]	USA	To assess the effectiveness of ACT Parents Raising Safe Kids (ACT-PRSK) in reducing child bullying	72 parents	RCT	ACT-PRSK program (Duration not specified)	**Questionnaire**: Revised Olweus’ Bully/Victim Questionnaire **Outcome**: Significant reduction in bullying behavior (*p* < 0.05).	Intervention effectively reduced aggressive behaviors in children.
5.	(Tosun & Mihci, 2020) [38]	Türkiye	To evaluate digital parenting education programs for parents in preventing bullying	231 parents	RCT	Digital Parenting Education (Duration not specified)	**Questionnaire**: Digital Parenting Attitude Scale **Outcome**: Increased public awareness of digital parenting concepts (*p* = 0.04).	Digital parenting education improved parents’ attitudes and engagement in managing children’s online activities.
6.	(Kim et al., 2021) [39]	China	To examine the impact of early childhood parenting on preventing bullying in adolescents	280 parents	RCT	Early childhood parenting training (10 years follow-up)	**Questionnaire**: Stattin and Kerr Parental Control & Monitoring Questionnaire **Outcome**: Parenting interventions significantly reduced bullying behavior (*p* = 0.026).	Early childhood interventions had long-term benefits in reducing adolescent bullying.
7.	(Díaz-Caneja et al., 2021) [40]	Spain	To assess the efficacy of a web-enabled, school-based bullying prevention intervention	1,882 children, parents, and teachers	RCT	Web-Enabled School-Based Prevention Program (12 weeks)	**Questionnaire**: Digital Parenting Attitude Scale **Outcome**: Effective in preventing bullying behavior (*p* < 0.05).	Online-based interventions showed promising results in school bullying prevention.
8	(Lester et al., 2017) [33]	Australia	To evaluate whole-school bullying interventions	1,400 parents and carers of Grades 2, 4, and 6 students	RCT	Whole-School Bullying Intervention (Duration not specified)	**Questionnaire**: Perceived Influence on Child’s Response to Being Bullied **Outcome**: Parents reported improved communication with children about bullying (*p* < 0.001).	Parent-focused interventions enhanced family discussions about bullying.
9	(Cross et al., 2018) [34]	Australia	To evaluate parent involvement in whole-school actions to reduce bullying	3,200 parents of Grade 2, 4, and 6 students	RCT	Whole-School Bullying Prevention Program (22 months)	**Questionnaire**: Parents’ Attitudes Towards Bullying Behavior Scale **Outcome**: Increased parental self-efficacy in addressing bullying (*p* < 0.05).	Parental involvement in school-wide interventions increased their awareness and engagement in bullying prevention.
10	(Farmer et al., 2017) [37]	New Zealand	To assess the effect of playground environment changes on bullying prevention	840 children, 635 parents, 90 teachers	RCT	School Playground Environment Changes (2 years)	**Questionnaire**: Peer Relations Assessment Questionnaires-Revised (PRAQ-R) **Outcome**: Reduced bullying behavior (difference score: 0.20; 95% CI: 0.06–0.34; *P* = 0.009).	Changing playground environments promoted social interaction and reduced bullying incidents.

### Identification of nursing-led, family-based interventions

The review identifies various nursing-led, family-based interventions that focus on involving families in the process of reducing bullying among children and adolescents. These interventions are designed to address the issue from a holistic perspective by engaging not only the child but also their immediate family members [37]. Examples of such interventions include educational programs aimed at increasing awareness about bullying, counseling services that support both children and parents, family-based workshops to enhance skills in managing bullying, and parent-child communication training to improve dialogues about bullying issues [25, 32]. Nurses play a pivotal role in leading these interventions by coordinating activities, offering support, and ensuring that both the child and family are equipped with the tools and strategies necessary to address bullying [35]. These nursing-led initiatives ensure that interventions are tailored to the family unit, recognizing the importance of home environments in shaping children’s behavior and responses to bullying [36].

### Mapping of intervention approaches

The interventions identified in this review can be classified into several distinct approaches, each designed to address different facets of bullying prevention. The authors found that there are three types, namely cooperation between schools and families, family-based education, and improving parenting patterns.

#### Cooperation between schools and families

Research shows that there are three types of interventions that are effective in overcoming the problem of bullying in the school environment. The first is cooperation between schools and families [32]. This involves active collaboration between the school and parents in identifying, preventing and dealing with cases of bullying [25]. Through this collaboration, schools and families share information about children’s behavior, discuss coping strategies, and provide the support and resources needed to effectively address bullying issues [33]. Collaboration between schools and families is an important foundation in creating a safe and responsive school environment [34, 40].

#### Family-based education

The second is family-based education, which is an intervention that focuses on providing education and training to parents about the signs of bullying, how to respond to it, and the importance of strengthening relationships with their children [35]. Through educational programs such as workshops, seminars, or counseling sessions, parents are given a better understanding of the dynamics of bullying and how they can help their children deal with it. By strengthening the role of parents as a source of support and mentors for their children, this intervention can reduce the risk of bullying behavior among children. Additionally, enhancing both parents’ and children’s resilience can help mitigate the negative impact of bullying, enabling them to develop coping strategies and emotional strength in facing such experiences [37].

#### Improving parenting patterns

third, improving parenting patterns to prevent bullying behavior is another type of intervention that is widely researched. This intervention aims to change parenting patterns that may support or allow bullying behavior to occur. This can include building positive communication skills, modeling good behavior, and applying clear and consistent limits to aggressive behavior [36]. Efforts to provide support and guidance to parents in developing positive and supportive parenting patterns, this intervention can help prevent the emergence of bullying behavior among children [38]. The research results show that cooperation between schools and families, family-based education, and improving parenting patterns are three types of interventions that are effective in overcoming the problem of bullying in the educational environment [39].

### Effectiveness and outcomes

The effectiveness of the identified interventions varies, with some demonstrating significant success in reducing bullying behaviors, particularly when both educational and family-centered approaches are combined. Studies indicate that interventions involving family participation have led to positive outcomes, such as improved emotional regulation among children, better conflict resolution skills, and stronger family relationships [34]. For instance, in one study, children whose families participated in the intervention reported a reduction in bullying incidents, with their emotional distress significantly decreased [38]. Additionally, increased awareness of bullying dynamics among parents contributed to better communication and support at home, which further supported the child’s ability to cope with bullying. Moreover, these interventions were also found to have a ripple effect, as family members became more proactive in preventing bullying behaviors, resulting in a more protective environment for the child [39].

### Challenges and barriers

Despite the promising outcomes of nursing-led, family-based interventions, several challenges hinder their successful implementation. One of the primary barriers is the lack of resources and training for nurses, who are often responsible for leading and facilitating these programs. Inadequate preparation can lead to ineffective delivery and a failure to engage families [36]. Additionally, cultural differences in family dynamics and parenting styles may affect how families participate in these interventions [39]. For example, some families may not fully embrace the idea of family-based interventions due to cultural perceptions of bullying or the role of nurses in family matters. Logistical issues, such as scheduling conflicts and difficulty coordinating between families and program facilitators, also pose significant barriers. Lastly, interventions must be customized to accommodate diverse family structures and socioeconomic backgrounds to ensure broad accessibility and participation [33, 40].

### Gaps and future directions

While existing interventions show promise, there are notable gaps that need to be addressed in future research. A significant limitation is the lack of long-term studies that evaluate the sustained effectiveness of nursing-led, family-based interventions. Most studies focus on short-term outcomes, leaving a gap in understanding the long-term impact of these interventions on bullying behaviors and family dynamics. Furthermore, there is a need for more research on the adaptability of these interventions across different cultural contexts, particularly in rural or marginalized communities where resources may be scarce. The review suggests that more randomized controlled trials (RCTs) are needed to provide robust evidence on the effectiveness of these interventions. Future studies should also explore how to increase family engagement, especially in communities where participation in such programs is low due to social, cultural, or logistical reasons.

## Discussion

The research results show that family-based intervention is an intervention that involves the family and is effective in reducing bullying behavior in children and adolescents (p value < 0.05). This shows that family involvement is important in shaping children’s character and preventing bullying behavior. The authors found that there are three types of family-based intervention, namely collaboration between school and family, family-based education, and improving parenting patterns.

Research on family-based interventions led by nurses has shown that this approach can improve the quality of care and patient health outcomes. Several studies have demonstrated that involving families in the care process can accelerate patient recovery and enhance their understanding of the health conditions they experience [15, 41]. For example, in the care of patients with chronic diseases, families involved in the care process play a crucial role in continuously monitoring the patient’s health, providing emotional support, and ensuring medication adherence [42]. This evidence shows that family-involved care can improve the effectiveness of care and the quality of life for patients.

Family-based interventions have become a focal point in nursing because families have a significant influence on the physical and psychosocial well-being of patients. These interventions not only provide education to families about the patient’s medical condition but also improve communication and decision-making processes between the patient, family, and the care team [43]. Families involved in the care process can reduce the levels of anxiety and stress experienced by patients because they feel more empowered and prepared to face the challenges of caregiving [44]. Furthermore, family-based approaches can enhance the patient’s quality of life through the reinforcement of social support provided by family members.

One of the most commonly used tools is the Olweus’ Bully/Victim Questionnaire [32, 36]. This questionnaire is widely recognized for its robustness in identifying both victims and perpetrators of bullying. Its advantage lies in its detailed assessment of bullying frequency, types, and the school environment, making it highly suitable for evaluating interventions aimed at reducing bullying behavior [45]. Another frequently used tool is the Digital Parenting Attitude Scale [38, 40]. This scale is particularly relevant in the context of cyberbullying and digital parenting interventions, as it measures parental attitudes toward digital media use and their role in preventing online harassment [46]. The increasing use of digital technology in children’s lives makes this instrument highly relevant for evaluating modern anti-bullying strategies. The Stattin and Kerr Parental Control and Monitoring Questionnaire [39]. This tool is designed to assess how well parents are aware of their children’s social activities, including involvement in bullying, both as perpetrators and victims. Its advantage is that it captures parental involvement and supervision, which are critical components of family-centred interventions [47].

The role of nurses in family-based interventions is crucial, as nurses are the ones who can coordinate communication between families and the medical team, ensuring that the care provided meets the needs of both the patient and the family. Nurses are responsible for educating families about patient care, providing emotional support, and teaching the skills needed to care for the patient at home [22]. Nurses also act as counselors who can help families cope with the psychological challenges that arise during the caregiving process. Through family-based approaches, nurses not only focus on the physical care of the patient but also support deeper psychosocial aspects, creating a more holistic and comprehensive care environment [48].

Family communication patterns, social support provided, and the level of supervision carried out by parents are several elements of family dynamics that significantly influence teenagers’ tendencies to engage in bullying behavior [49]. Families that implement open communication patterns and facilitate open discussions about social and emotional issues tend to have adolescents who are less likely to engage in bullying behavior [50]. In addition, social support provided by family members, such as listening and paying attention, also plays an important role in reducing the tendency for bullying behavior [15]. The appropriate level of supervision from parents regarding teenagers’ activities and interactions in the family environment also has a positive impact in suppressing bullying behavior [51]. This evidence confirms the strong link between family dynamics and the level of success of interventions in overcoming bullying behavior in adolescents [52, 53].

The challenges faced in implementing Interventions focused on Family to overcome bullying behavior in adolescents include several complex aspects. One is the difficulty in mobilizing parental participation in intervention programs, especially when they have busy schedules or lack awareness of the importance of their role in shaping children’s behavior [54]. In addition, the stigma or shame associated with admitting that their child is involved in bullying behavior can also be an obstacle to getting adequate family support [37, 55]. To overcome these challenges, it is necessary to take a holistic and inclusive approach involving communities, schools and mental health service providers to strengthen support and provide assistance to parents [56].

However, there are also significant opportunities for continued research and practice in the development and implementation of anti-bullying interventions involving families [57]. One opportunity is the development of digital technology and online platforms that can be used to provide resources, support and information to parents in supporting their children in overcoming bullying problems [58]. In addition, further research can explore the potential for collaboration between educational institutions, community organizations, and government agencies to create integrated and sustainable intervention programs [59, 60]. By leveraging technology and cross-sector collaboration, Interventions focused on Family can become more accessible, effective, and relevant to community needs [37, 61]. In this way, the challenges faced can be overcome while taking advantage of these opportunities to increase the effectiveness of anti-bullying interventions involving families.

Collaboration between schools and families is a type of family-based intervention that is effective in reducing bullying behavior in children and adolescents. Previous research shows the important role of both in preventing bullying, but the focus is more limited to one environment [62, 63]. The advantage of this intervention lies in the holistic integration between formal education at school and the influence of the home environment [64, 65]. However, the challenge is that coordination between the two institutions is often difficult and differences in values and beliefs can hinder consistent strategy implementation. Collaboration between schools and families promises a significant reduction in bullying behavior, but requires commitment, coordination and deep understanding to achieve success in implementation [34, 66].

Family-based education is a type of family-based intervention that is effective in reducing bullying behavior in children and adolescents. Previous research has demonstrated the important role of families in preventing and addressing bullying, but there is variation in the approaches used [67]. The advantage of family-based education lies in its ability to involve families directly in providing education and skills to children regarding conflict resolution, emotional management and healthy communication [68, 69]. In addition, this approach allows for more sustainable program implementation because it actively involves parents in changing their child’s behavior. However, challenges include ensuring parental participation and commitment, as well as the suitability of the program to the needs and values of diverse families [70]. Implementation of this intervention requires a sensitive and comprehensive approach to family dynamics as well as ongoing support from the school and community [71].

Structured workshops that focus on bullying prevention, positive parenting, and cyberbullying awareness provide parents with essential knowledge and practical skills to support their children effectively [32]. These workshops help improve parent child communication, foster empathy, and reinforce consistent discipline strategies that discourage aggressive behavior. Parent education programs not only reduce children’s vulnerability to peer victimization but also increase parents’ confidence in managing challenging social issues [72]. When conducted in collaboration with schools and child psychologists, such programs can ensure evidence-based content that is developmentally appropriate and sensitive to the emotional needs of both parents and children [73]. This holistic and collaborative approach aligns with an ecological framework for bullying prevention, which underscores the importance of engaging multiple systems family, school, and community in coordinated intervention strategies [74].

Improving parenting patterns is one type of family-based intervention that is effective in reducing bullying behavior in children and adolescents [75]. An emphasis on improving parenting styles in the context of bullying prevention offers its own advantages, including its ability to improve relationships between parents and children, improve communication skills, and form a better understanding of children’s emotions [76]. Efforts to strengthen family relationships and provide responsive and targeted parenting, this intervention can reduce the risk of children being involved in bullying behavior [77]. However, challenges faced include the need for long-term commitment from parents, as well as adapting intervention strategies to varying family and cultural contexts [78]. The importance of collaboration between psychologists, educators and public health workers in supporting the implementation of interventions to improve parenting patterns as a holistic and sustainable effort to overcome the problem of bullying behavior in children and adolescents [79].

There is consistency in the findings that interventions that actively involve families are able to provide significant protection for adolescents from exposure to bullying behavior [80]. Through in-depth research, it was found that parental involvement, increasing intra-family communication, and providing adequate emotional support were key elements in the success of the intervention [81]. Overall, these findings provide strong evidence of the important role of families in reducing bullying behavior in adolescents, and provide a solid foundation for the development of further intervention strategies that utilize potential family resources.

### Implications for practice

The findings of this scoping review suggest that nurses have an essential role to play in developing, implementing, and evaluating family-based interventions aimed at reducing bullying among children and adolescents. To improve practice, it is crucial that nurses receive training not only in bullying prevention strategies but also in understanding family dynamics and how to engage families effectively. Healthcare institutions must prioritize the integration of these interventions into existing health and community services, ensuring that nurses are adequately supported and resourced. Collaboration between nurses, families, schools, and other professionals is necessary to create a supportive environment for children at risk of bullying. Furthermore, the review emphasizes the importance of sustainability and the need for continued professional development to keep pace with emerging trends in bullying prevention. In this context, nurses play a crucial role in strengthening the resilience of children and their families who experience bullying. Through educational programs, emotional support, and counseling interventions, nurses can help families develop effective coping mechanisms, enhance self-efficacy, and foster a supportive home environment. By equipping both parents and children with the necessary skills to navigate and respond to bullying incidents, nurses contribute to long-term prevention efforts and improve overall psychological well-being. Structured workshops can be organized for parents on topics such as bullying prevention, positive parenting strategies, and cyberbullying awareness. These workshops should be conducted in collaboration with the institution and child psychologists to ensure evidence-based content and holistic support for families. Furthermore literature can be added on policies and guidelines set by the institution to reduce the bullying reports.

### Limitations

Limitations in this scoping review include the design used in the selection of articles is RCT and quasi experiment, so it cannot describe the results of research with other designs. In addition, the year of publication was also limited to the last 10 years, so data outside the year of publication was not described in this study. Lastly, scoping reviews tend to produce less in-depth summaries or more superficial analysis, which may not illustrate the effectiveness of Interventions focused on Family.

## Conclusion

The findings reveal that 10 articles discuss nurse led family-focused interventions, which have shown significant potential in addressing bullying. These interventions primarily focus on enhancing parenting patterns, providing family-based education, and fostering collaboration between schools and families. Nurses play a key role in coordinating these interventions by offering education, emotional support, and guidance to families, as well as facilitating communication between families and other professionals. Interventions focused on Family show significant potential in addressing bullying behavior in children and adolescents, with an emphasis on improving parenting patterns, family-based education, and collaboration between schools and families. The challenges of these interventions are long-term commitment from parents and adapting strategies for diverse family contexts. It offers a holistic and sustainable approach that can improve family relationships, communication skills and understanding of children’s emotions. Activities undertaken in Interventions focused on Family are education, role-playing, counseling, managing conflict, and conducting online and offline assessments.

Recommendations for future research include more in-depth research on the factors that influence the successful implementation of the intervention, the best strategies to engage parents and schools, and the long-term impact of this intervention on child and adolescent well-being. In addition, further research is also needed to determine the effectiveness of Interventions focused on Family to reduce bullying behavior in children and adolescents with a systematic review design and meta-analysis.

## Data Availability

All data generated or analysed during this study are included in this published article.
